# Adverse events in patients with ankylosing spondylitis treated with TNF inhibitors: a cross-sectional study

**DOI:** 10.1007/s11096-019-00859-7

**Published:** 2019-06-06

**Authors:** Jakub Wroński, Piotr Fiedor, Piotr Głuszko

**Affiliations:** 1 Department of Rheumatology, National Institute of Geriatrics, Rheumatology and Rehabilitation, Spartańska 1, 02-637 Warsaw, Poland; 2 Department of General and Transplantation Surgery, Medical University of Warsaw, Nowogrodzka 59, 02-006 Warsaw, Poland

**Keywords:** Adverse events, Ankylosing spondylitis, Retrospective study, Safety, Tumor necrosis factor inhibitors

## Abstract

*Background* Although TNF inhibitors are well established in ankylosing spondylitis treatment, the majority of studies on TNF inhibitors safety have been performed in rheumatoid arthritis patients. Meanwhile, it seems that TNF inhibitors in ankylosing spondylitis may present a better safety profile than we thought. *Objective* The aim of our study was to retrospectively investigate the occurrence of adverse events in ankylosing spondylitis patients treated with TNF inhibitors. *Setting* A single referral center in Poland. *Methods* Detailed medical history of ankylosing spondylitis patients was obtained during the interview with the patient and by reviewing electronic medical records. Patients treated with TNF inhibitors and patients without TNF inhibitors treatment were compared. *Main outcome measure* The incidence of adverse events during the 3 months period before the interview. *Results* A total of 150 patients, 103 in the treatment group and 47 in the control group, were included in the study. There were no differences in the incidence of adverse events, serious adverse events, infections and opportunistic infections between both groups. However, in the treatment group, noninfectious adverse events were significantly less frequent than in control group (RR 0.39, 95% CI 0.23–0.66), with abdominal pain as the most common noninfectious adverse event (RR 0.20, 95% CI 0.07–0.63). The differences in incidence rates of specific infections were not significant, except acute infectious diarrhea which also was less frequent in patients treated with TNF inhibitors (RR 0.17, 95% CI 0.03–0.85). The female gender was significantly associated with any adverse event occurrence (OR 2.36, 95% CI 1.15–4.83). *Conclusion* TNF inhibitors show a good safety profile in ankylosing spondylitis patients.

**Electronic supplementary material:**

The online version of this article (10.1007/s11096-019-00859-7) contains supplementary material, which is available to authorized users.

## Impacts on practice

- TNF inhibitors in ankylosing spondylitis patients may be safer than previously thought, based on prior studies performed in rheumatoid arthritis patients.
- Treatment with TNF inhibitors in ankylosing spondylitis patients may be better tolerated than standard first-line treatment with NSAIDs.

## Introduction

Ankylosing spondylitis (AS) is a chronic progressive auto-inflammatory disease predominantly affecting the axial skeleton, occurring in about 0.23% of the European population [1]. The progressive ossification of vertebral column resulting from the chronic inflammation leads gradually to irreversible loss of spinal mobility, and as a result, disability. Treatment of AS is targeted to improve symptoms, functionality and prevent progression of the disease. First line therapy for AS is physical exercise together with the continuous use of nonsteroidal anti-inflammatory drugs (NSAIDs) in well-responding patients otherwise symptomatic [2]. Such treatment is relatively ineffective (ASAS20 response is achieved only in about 57% of patients treated with NSAIDs [3]) and associated with multiple risks (cardiovascular, gastrointestinal, renal) that should be considered. Fortunately, targeted therapy with TNF inhibitors (TNFi) has been introduced into clinical practice. TNFi proved to be effective in AS patients in improving clinical symptoms [4] and in long-term use they may prevent structural damage progression [5]. Unfortunately, given the high cost of TNFi therapy, currently, there are no guidelines on how to sustain remission of the disease without continuous TNFi treatment. After discontinuation of TNFi high percentage of patients experience flares in a short time [6]. Up to date, long-term use of TNFi in AS patients remains necessary, which raises concerns about the long-term safety of TNFi in AS patients. Although TNFi are well established in AS treatment (first TNFi have been approved to use in AS by FDA in 2003), the majority of studies on their safety have been performed in rheumatoid arthritis (RA) patients. Until recently, there were very few real-life studies regarding TNFi adverse effects in AS patients. Meanwhile, it seems that TNFi may present a better safety profile in AS than in RA [7].

### Aim of the study

The aim of our study was to retrospectively investigate and compare the occurrence of adverse events (AE) in AS patients treated with and without TNFi.

### Ethics approval

The study protocol has been approved by the hospital bioethics committee (approval number KBT-6/6/2016). All participants have signed informed consents for inclusion in the study. The study was conducted according to the Declaration of Helsinki.

## Methods

The study was conducted at a rheumatology institute in Poland between September 2016 and March 2018. Patients with AS (fulfilling the 1984 modified New York criteria) were recruited from inpatient and outpatient clinics. All AS patients treated with TNFi were treated according to the national therapeutic program (Supplementary Table 1) and before the beginning of TNFi treatment had to have active disease (BASDAI value ≥ 4 or ASDAS value ≥ 2.1 for at least 4 weeks) despite the treatment with at least two NSAIDs at maximal doses for at least 4 weeks each. Detailed medical history was obtained during the interview with the patient and, for laboratory data and if not obtained during the interview, by reviewing electronic medical records.

Data regarding patients demographics and characteristics, including age, sex, the presence of HLA-B27 antigen, duration of the disease (from the moment of diagnosis and from the first symptoms), prior and current use of classical disease-modifying antirheumatic drugs (cDMARDs) and TNFi, as well as current use of other medications were collected. Risk factors for infections and malignancies were evaluated. The occurrence of AE, serious adverse events (SAE), infections, serious infections, opportunistic infections, noninfectious AE and discontinuations due to AE in the last 3 months was reported, including information about the type, location, and required treatment. The Common Terminology Criteria for Adverse Events Version 5.0 were used for AE grading [8]. Treatment efficacy—the disease activity (measured with BASDAI and ASDAS), patients functioning (measured with BASFI) and markers of inflammation (CRP, ESR) were assessed.

Retrospective analysis of collected data was performed. TNFi treated patients (treatment group) and patients without TNFi treatment (control group) were compared. Compatibility with the normal distribution of data obtained was assessed using the Shapiro–Wilk test. The significance of observed differences was measured using the Student’s *t* test for data with normal distribution and the Mann–Whitney U test for data with non-normal distribution. For categorical data, the Pearson’s Chi squared test or the Fisher’s exact test (for tables with values less than 5) were performed. For AE relative risks (RR) and corresponding 95% confidence intervals (95% CI) were calculated. Logistic regression and odds ratio (OR) with 95% CI were used to identify predictive factors associated with different types of AE and good clinical response. The final multivariate model was created by the stepwise-backward method, variables from the univariate analysis with a likelihood-ratio p-value less than 0.1 were used. Statistical significance was set at *p* < 0.05. Statistical analysis was performed using Statistica 13.1 software.

## Results

A total of 150 patients, 103 in the TNFi treatment group and 47 in the control group, were included in the study. The demographics and characteristics of both groups are shown in Table 1. TNFi treatment group had a significantly longer duration of the diagnosed disease than patients without TNFi treatment (mean 11.76 years vs. 8.42 years respectively), but there was no difference in the time from symptoms onset. Patients without TNFi used significantly more NSAIDs compared to TNFi treatment group (82.98% vs. 50.49% respectively), more NSAIDs at maximum doses (66.67% vs. 35.29%) and more of them used NSAIDs continuously (66.67% vs. 18.18%), whereas patients treated with TNFi used the same NSAIDs (without the need for changing the NSAID) for longer time (mean duration of treatment 2086 days vs. 985 days). Data regarding the specific NSAIDs used and the maximum doses are presented in Supplementary Table 2. There were no differences in concomitant use of cDMARDs and glucocorticoids, as well as the previous use of cDMARDs and TNFi treatment. Previous TNFi treatment was discontinued due to AE in 2 cases (recurrent infections in one case after adalimumab and allergic reaction with skin changes in the second case after infliximab), due to treatment inefficiency in 3 cases, and due to the end of the treatment program in 8 cases. One patient had also basal-cell carcinoma during treatment with etanercept, but treatment was not discontinued. In the treatment group, the most common TNFi was adalimumab (49.51%), followed by etanercept and biosimilar etanercept SB4 (together 27.18%), golimumab (17.48%), biosimilar infliximab CT-P13 (4.85%) and certolizumab (0.97%). The risk factors of infections and malignancy are listed in Supplementary Table 3, but except HPV infection history (more frequent in the control group) did not differ significantly between both groups.

**Table 1:** Patient demographics and characteristics

	TNFi treatment (n = 103)	No TNFi treatment (n = 47)	Difference
Age, mean ± SD	42.69 ± 12.42	45.87 ± 14.45	NS
Sex, number (%)			
Male	74 (71.8%)	28 (59.6%)	NS
Female	29 (28.2%)	9 (40.4%)	
BMI mean ± SD	25.78 ± 4.26	26.06 ± 4.29	NS
HLA-B27 positive number (%)	79 (76.70%)	37 (78.72%)	NS
Symptoms duration mean years ± SD	16.75 ± 10.03	15.44 ± 11.43	NS
First diagnosis mean years ago ± SD	11.76 ± 13.96	8.42 ± 9.97	*p* = 0.01583
TNFi treatment		–	–
Mean duration (days) ± SD	906.28 ± 849.38		
Number (%) of patients treated with:			
Adalimumab	51 (49.51%)		
Certolizumab	1 (0.97%)		
Etanercept	11 (10.68%)		
Biosimilar etanercept (Benepali)	17 (16.50%)		
Biosimilar infliximab (Remsima)	5 (4.85%)		
Golimumab	18 (17.48%)		
NSAIDs treatment number of patients (%)	52 (50.49%)	39 (82.98%)	*p* = 0.00016
Continuous use	10 (18.18%)	26 (66.67%)	*p* < 0.00001
Maximum dose	18 (35.29%)	26 (66.67%)	*p* = 0.00317
Mean duration of treatment (days) ± SD	2086.56 ± 2286.55	985.64 ± 1831.55	*p* = 0.00110
Concomitant DMARDs number of patients (%)	22 (21.36%)	15 (31.91%)	NS
Use of MTX	14 (13.59%)	7 (14.89%)	NS
Use of SSZ	10 (9.71%)	10 (21.28%)	NS
Use of MTX and SSZ	2 (1.94%)	2 (4.26%)	NS
Mean duration of treatment (days) ± SD	1879.67 ± 2402.95	1113.50 ± 1408.86	NS
Concomitant GCs number (%)	9 (8.74%)	6 (12.77%)	NS
Mean dose in mg of methylprednisolone ± SD	5.11 ± 4.51	10 ± 7.59	NS
Mean duration of treatment (days) ± SD	3389.75 ± 3399.77	555 ± 701	NS
Previous DMARDs history number (%)	79 (77.45%)	31 (65.96%)	NS
Mean number of previous DMARDs ± SD	1.58 ± 0.63	1.58 ± 0.67	NS
Previous TNFi history number (%)	9 (8.74%)	4 (8.51%)	NS
Laboratory parameters			
Mean AST ± SD	30.70 ± 19.88	37 ± 21.42	NS
Mean ALT ± SD	25.79 ± 9.80	28.70 ± 12.24	NS
Mean GFR ± SD	124.48 ± 37.28	113.35 ± 38.36	NS
Mean Hb ± SD	14.47 ± 1.46	13.56 ± 1.9	*p* = 0.00902
Mean WBC ± SD	7.14 ± 2.30	7.28 ± 2.5	NS
Mean PLT ± SD	262 ± 74.03	308.91 ± 97.4	*p* = 0.00064

*DMARDs* disease modifying antirheumatic drugs, *GCs* glucocorticoids, *NS* not significant, *NSAIDs* nonsteroidal anti-inflammatory drugs, *TNFi* TNF inhibitors

The occurrence of AE is presented in Table 2. There were no differences in the incidence of any AE, SAE, infections and opportunistic infections between both groups. However, in the treatment group noninfectious AE were significantly less frequent than in patients without TNFi treatment—with RR of 0.39 (95% CI 0.23–0.66). Table 3 contains the detailed list of all AE and their RR. There was only one SAE—persistent tachycardia after adalimumab administration, requiring hospitalization in the emergency department. The most common infections were upper respiratory tract infections. There were 5 opportunistic infections in the treatment group, 4 herpes simplex cases and 1 case of chronic furunculosis, in contrast to only one case of herpes simplex in the control group. However, the differences in incidence rates of specific infections were not significant, except acute infectious diarrhea which was significantly less frequent in TNFi treatment group (RR 0.17, 95% CI 0.03–0.85). The most common noninfectious AE was abdominal pain and was also significantly less frequent in the treatment group (RR 0.20, 95% CI 0.07–0.63). Some paradoxical AE occurred during the study—1 case of new onset of psoriasis during etanercept treatment and 2 cases of uveitis during golimumab treatment. No patient needed to discontinue treatment due to AE. The female gender was significantly associated with any AE occurrence (OR 2.36, 95% CI 1.15–4.83, *p* = 0.0283). No other covariates (listed in Table 1 and Supplementary Table 3) were significant with any AE, noninfectious AE and infections occurrence in multivariate logistic regression. There were no differences in the rate of AE between different TNFi.

**Table 2:** Adverse events

	TNFi treatment Number of patients (%)	No TNFi treatment Number of patients (%)	
Any AE	47 (45.63%)	27 (57.45%)	
Grade: mild	26 (25.24%)	18 (38.30%)	
Moderate	20 (19.42%)	7 (14.89%)	
Severe	1 (0.97%)	0	
Life-threatening	0	0	
Death related to AE	0	0	
Serious AE	1 (0.97%)	0	
Infections	38 (38%)	15 (34.09%)	
Serious infections	0	0	
Opportunistic infections	5 (4.85%)	1 (2.27%)	
Noninfectious AE	15 (14.56%)	15 (31.91%)	
Discontinuation due to AE	0	0	

	Total events	Total events	Relative risk (95% CI)
Any AE	75	40	0.86 (0.72, 1.01)
Serious AE	1	0	1.30 (0.05, 31.26)
Infections	57	19	1.28 (0.88, 1.88)
Opportunistic infections	5	1	2.14 (0.26, 17.76)
Noninfectious AE	18	21	0.39 (0.23, 0.66)*

*AE* adverse events, *TNFi* TNF inhibitors

*p = 0.0005

**Table 3:** Occurrence of non-infectious and infectious AE

	TNFi treatment number of events	No TNFi treatment number of events	Relative risk (95% CI)
Infections			
Upper respiratory tract infection	46	13	1.51 (0.91, 2.50)
Herpes simplex	4	1	1.71 (0.20, 14.86)
Acute infectious diarrhea	2	5	0.17 (0.03, 0.85)*
Urinary tract infection	1	0	1.30 (0.05, 31.26)
Lower respiratory tract infection	1	0	1.30 (0.05, 31.26)
Genital tract infection	1	0	1.30 (0.05, 31.26)
Skin Infection	1	0	1.30 (0.05, 31.26)
Otitis media	1	0	1.30 (0.05, 31.26)
Non-infectious AE			
Abdominal pain	4	9	0.20 (0.07, 0.63)**
Elevated transaminases	3	3	0.46 (0.10, 2.17)
Leukopenia	3	2	0.68 (0.12, 3.91)
Skin changes	0	3	0.07 (0.004, 1.25)
Diarrhea	0	2	0.09 (0.005, 1.89)
Uveitis	2	1	0.91 (0.08, 9.82)
Psoriasis	1	0	1.39 (0.06, 33.37)
Nausea	1	0	1.39 (0.06, 33.37)
Dizziness	1	0	1.39 (0.06, 33.37)
Tachycardia	1	0	1.39 (0.06, 33.37)
Periodontitis	1	0	1.39 (0.06, 33.37)
Colitis	0	1	0.15 (0.006, 3.71)

*AE* adverse events, *TNFi* TNF inhibitors

*p = 0.0306, **p = 0.0055

The therapy efficacy is presented in Table 4. TNFi treatment group had significantly better BASDAI (mean 3.23 vs. 5.26), ASDAS (mean 2.16 vs. 3.41) and BASFI (mean 2.92 vs. 4.08) scores compared to the control group, as well as mean values of inflammatory markers—CRP (10.39 vs. 13.54) and ESR (14.33 vs. 24.53). Also indirect indicators of inflammation—mean platelet count and mean hemoglobin values were significantly better in the treatment group. In TNFi treated group, 54% of patients achieved remission or low disease activity compared to less than 11% in the control group. In the multivariate logistic regression analysis, no other factors than TNFi treatment (OR 8.30, 95% CI 2.18–31.60) were significantly associated with remission or low disease activity.

**Table 4:** Therapy efficacy

	TNFi treatment (n = 103) Mean ± SD	No TNFi treatment (n = 47) Mean ± SD	Difference
BASDAI	3.23 ± 1.67	5.26 ± 1.96	*p* < 0.00001
Axial pain	3.67 ± 1.92	6.04 ± 2.26	*p* < 0.00001
Peripheral joints pain/swelling	2.79 ± 2.08	4.45 ± 2.75	*p* = 0.00021
Enthesitis	2.53 ± 2.00	3.75 ± 2.85	*p* = 0.00957
Morning stiffness duration	3.02 ± 2.07	6.04 ± 3.44	*p* < 0.00001
Morning stiffness level	3.23 ± 1.94	6.04 ± 2.73	*p* < 0.00001
Fatigue	4.01 ± 2.15	6.04 ± 2.33	*p* < 0.00001
ASDAS	2.16 ± 0.94	3.41 ± 0.99	*p* < 0.00001
Patient global assessment	3.37 ± 1.84	5.85 ± 2.42	*p* < 0.00001
BASFI	2.92 ± 2.04	4.08 ± 2.28	*p* = 0.00381
Putting on socks	2.39 ± 2.43	3.89 ± 3.04	*p* = 0.00521
Bending forward	3.45 ± 2.64	4.92 ± 3.17	*p* = 0.00643
Reaching up	2.52 ± 2.50	3.51 ± 3.22	NS
Getting up from a chair	2.36 ± 2.79	3.53 ± 3.22	*p* = 0.04035
Getting up off the floor from lying	3.10 ± 2.44	5.11 ± 2.71	*p* = 0.00003
Standing unsupported	3.15 ± 3.01	4.23 ± 3.09	*p* = 0.04894
Climbing 12–15 steps	2.30 ± 2.60	3.32 ± 3.12	NS
Looking over your shoulder	4.35 ± 3.25	5.40 ± 3.18	NS
Doing physically demanding activities	3.57 ± 2.45	4.87 ± 3.13	*p* = 0.01057
Doing a full day activities	2.01 ± 1.94	2.04 ± 2.59	NS
ESR	14.33 ± 15.69	24.53 ± 23.95	*p* = 0.00692
CRP	10.39 ± 26.69	13.54 ± 13.59	*p* < 0.00001
Remission or low disease activity number (%)	56 (54.37%)	5 (10.64%)	*p* < 0.00001

*ASDAS* Ankylosing Spondylitis Disease Activity Score, *BASDAI* Bath Ankylosing Spondylitis Disease Activity Index, *BASFI* Bath Ankylosing Spondylitis Functional Index, *NS* not significant, *TNFi* TNF inhibitors

## Discussion

TNFi have been successfully used for the treatment of AS for 15 years. However, it has only recently been suggested that TNFi may have a better safety profile in AS when compared to their known safety profile in RA. Our study is the first study evaluating the safety of TNFi in the Polish population of AS patients, and one of the few observational studies on this subject in the world. Our results show good safety profile of TNFi in AS patients and are in accordance with available data. All meta-analyses of randomized controlled trials (RCTs) of AE in AS patients performed up to date demonstrated no significant difference in serious AE [4, 9–12], infections [11, 13], serious infections [9, 11–14], or malignancies [12, 15] rates in a group of AS patients treated with TNFi. Although one meta-analysis showed increased risk of overall AE in TNFi treated group compared to placebo (RR 1.22, 95% CI 1.12–1.33), it was probably due to increased risk of injection-site reactions after TNFi (RR 2.93, 95% CI 2.02–4.23), as there was no increase in other types of AE [11].

The most interesting result of our study is the lack of increased occurrence of infections in TNFi treated AS patients. Infections, including serious infections, are the most important and best established adverse effects of TNFi. It is not surprising as TNF is the key mediator of the host response to infection [16]. Increase of infection and serious infections risk after TNFi was confirmed in both meta-analysis of RCTs [17, 18] and observational studies [19, 20] in RA patients, but not in RCTs of AS patients. The only observational study comparing infection rates in AS patients with and without TNFi treatment, performed in Toronto clinic, showed no significant difference in the incidence rate of any infections and serious infections (respectively 19 and 1.5 per 100 patient-years in TNF treatment group) [21]. The observed differences between the number of infections after TNFi in patients with RA and AS may be due to several factors. Patients with AS are usually younger and use fewer cDMARDs and glucocorticoids prior to and during the treatment with TNFi compared to RA patients. However, the main difference could be the difference in pathophysiology of both diseases, as RA is characterized by increased baseline risk of infections (hazard ratio (HR) 1.70, 95% CI 1.42–2.03) and serious infections (HR 1.83, 95% CI 1.52–2.21) [22]. Previous studies showed that only specific types of infection may have an increased incidence in AS after TNFI, including tuberculosis [23] and herpes zoster [24]. In our study, there were no cases of either, probably due to short reporting period and a relatively small population, as both types of infections are rare. What is more, in Poland all patients before starting TNFi treatment have the interferon-γ release assay done, and in the case of latent tuberculosis, all patients receive chemoprophylaxis. It was shown in RA patients treated with TNFi that chemoprophylaxis in patients with latent tuberculosis decreased the risk of tuberculosis infection to similar levels as in patients without latent tuberculosis [25].

In our study not only TNFi treatment group did not have increased rates of AE, SAE, infections and opportunistic infections, but also had significantly less noninfectious AE, due to less complains about abdominal pain. This is not surprising, as the treatment group used significantly less often NSAIDs, fewer NSAIDs at maximum doses and they more often used NSAIDs only on demand. The treatment group had also fewer cases of acute infectious diarrhea. It may be due to the fact that NSAIDs are known risk factor for acute diarrhea occurrence [26].

Current knowledge about adverse effects in AS patients after TNFi is based mainly on data from studies of RA patients, in which TNFi seem to have a different safety profile. Our study adds evidence to support the opinion that TNFi in AS patients are not only effective but also safer than we thought. The strength of our study is the number of covariates analyzed. The biggest limitation is the cross-sectional character of the study and a relatively small sample size. Lack of statistically significant differences in AE rates may be possible through lack of power, which was also slightly reduced by the difference in group size. With a short period of observation the study could only detect frequent AE, but not others that happens during the prolongate use of TNFi. Large, real-world cohorts with long-term prospective observation would be the most appropriate for studying adverse effects of TNFi in AS patients, as adverse effects in this particular group seem to be rare. We hope for population-based registries to study this issue.

## Conclusions

In our study, there were no differences in the incidence of any AE, SAE, infections and opportunistic infections between AS patients treated with TNFi and control group. TNFi treatment group had significantly better BASDAI, ASDAS and BASFI scores compared to the control group. Moreover, the control group treated mainly with NSAIDs, had more noninfectious AE (abdominal pain) and more episodes of acute infectious diarrhea than patients treated with TNFi. TNFi are not only effective in treating AS but also present a good safety profile in AS patients, possibly better than in RA patients.

## Electronic supplementary material

Below is the link to the electronic supplementary material.

Supplementary material 1 (PDF 62 kb)
