# Supplementary material for: Adverse events in patients with ankylosing spondylitis treated with TNF inhibitors: a cross-sectional study
Source: Int J Clin Pharm. 2019 Jun 6;41(4):864–71. doi: 10.1007/s11096-019-00859-7 (PMC6677866; doi:10.1007/s11096-019-00859-7)
Supplement: Supplementary file 1 — Supplementary material 1 (PDF 62 kb) [file 11096_2019_859_MOESM1_ESM.pdf]

Inclusion criteria	Treatment regimen	Duration of treatment
Patients fulfilling the 1984 modified New York criteria of AS.<br>Active disease (BASDAI $\geq$ 4 or ASDAS $\geq$ 2.1 and axial pain $\geq$ 4cm in VAS and physician global assessment $\geq$ 5cm in VAS) for at least 4 weeks with no treatment change.<br>Failure of treatment with at least two NSAIDs at maximum tolerated doses, each used for at least 4 weeks.<br>In individual cases without fulfilling the above criteria if the patient is threatened with a disability or is threatened with his life.	Adalimumab – 40mg SC every 2 weeks<br>Certolizumab –200mg SC every 2 weeks after initial induction (400mg SC at weeks 0,2,4)<br>Etanercept – 50mg SC every week<br>Golimumab – 50mg SC every month<br>Infliximab – 5mg/kg IV every 8 weeks after initial induction (5mg/kg IV at weeks 0, 2, 6)	Between 15 and 18months if the low activity (BASDAI <3 or ASDAS <1,3) is obtained. Treatment can be repeated if after TNFi discontinuation there is a relapse of disease activity. If the relapse is earlier than in 12 weeks after the discontinuation – the treatment dosage and duration is determined by the attending physician.<br>Up to 6 months if the low activity (BASDAI <3 or ASDAS <1,3) is not obtained.<br>Up to 3 months if there is no improvement by 50% or 2 points in BASDAI or in ASDAS by 50% or 1.1 point
Exclusion criteria before treatment		Exclusion criteria during treatment
Contraindications specified in the summary of product characteristics and EULAR / ASAS recommendations.		The occurrence of side effects, which in the opinion of the attending physician are contraindications for the treatment with TNFi.

Supplementary Table 1: Summary of the Polish national therapeutic program for the treatment of ankylosing spondylitis with TNF inhibitors. AS – ankylosing spondylitis, ASAS - Assessment of SpondyloArthritis international Society, ASDAS - Ankylosing Spondylitis Disease Activity Score, BASDAI - Bath Ankylosing Spondylitis Disease Activity Index, EULAR - European League Against Rheumatism, NSAIDs - nonsteroidal anti-inflammatory drugs, TNFi – TNF inhibitors, VAS - visual analogue scale

	TNFi treatment *(n=52)*	No TNFi treatment *(n=39)*	Difference
number (%) of patients treated with:			
celecoxib	2 (3,85%)	3 (7,69%)	NS
diclofenac	29 (55,77%)	21 (53,85%)	NS
ketoprofen	7 (13,46%)	3 (7,69%)	NS
meloxicam	7 (13,46%)	8 (20,51%)	NS
naproxen	6 (11,54%)	3 (7,69%)	NS
nimesulide	1 (1,92%)	-	-
piroxicam	-	1 (2,56%)	-
The maximum daily dose for treating AS: celecoxib 400mg, diclofenac 150mg, ketoprofen 200mg, meloxicam 15mg, naproxen 1000mg, nimesulide – not recommended, piroxicam 20mg but not recommended as the first line treatment			

Supplementary Table 2: Use of specific nonsteroidal anti-inflammatory drugs and their recommended maximum daily dose in ankylosing spondylitis. AS – ankylosing spondylitis, NS – not significant, NSAIDs - nonsteroidal anti-inflammatory drugs, TNFi – TNF inhibitors

	TNFi treatment	No TNFi treatment	Difference
Smoking, number (%)<br>mean Pack-year ± SD	15 (14.71%)<br>11.94 ± 12.51	9 (19.15%)<br>10.06 ± 10.09	NS<br>NS
Alcohol consumption<br>mean standard drinks per week ± SD	1.9 ± 4.98	2.70 ± 9.10	NS
Diabetes, number (%)	1 (0.98%)	1 (2.13%)	NS
COPD or Asthma, number (%)	5 (4.85%)	2 (4.26%)	NS
Hematological treatment, number (%)	1 (0.98%)	2 (4.26%)	NS
Dialysis, number (%)	0	0	-
Liver failure, number (%)	0	0	-
Tuberculosis infection history, number (%)	6 (5.88%)	0	NS
Tuberculosis exposure history, number (%)	10 (9.80%)	4 (8.51%)	NS
HBV infection history, number (%)	2 (1.96%)	2 (4.26%)	NS
Opportunistic infections history, number (%)	5 (4.90%)	3 (6.38%)	NS
Influenza vaccination (last year), number (%)	6 (5.88%)	1 (2.13%)	NS
Pneumococcal vaccination (last 5 years), number (%)	1 (0.97%)	0	NS
Excessive UV exposure, number (%)	19 (18.63%)	10 (21.28%)	NS
Previous EBV infection, number (%)	5 (4.90%)	1 (2.13%)	NS
Previous HPV infection, number (%)	1 (0.98%)	4 (8.51%)	p=0.03450
Close family malignancy history, number (%)	47 (46.08%)	18 (38.30%)	NS
Malignancy history, number (%)	1 (0.98%)	1 (2.13%)	NS

Supplementary Table 3: Infection and malignancy risk factors. NS – not significant, TNFi – TNF inhibitors
